# Induction of *foxp3* during the Crosstalk between Antigen Presenting Like-Cells MHCII^+^CD83^+^ and Splenocytes CD4^+^IgM^−^ in Rainbow Trout

**DOI:** 10.3390/biology10040324

**Published:** 2021-04-13

**Authors:** Byron Morales-Lange, Ivan Nombela, María Del Mar Ortega-Villaizán, Mónica Imarai, Paulina Schmitt, Luis Mercado

**Affiliations:** 1 Grupo de Marcadores Inmunológicos en Organismos Acuáticos, Laboratorio de Genética e Inmunología Molecular, Instituto de Biología, Pontificia Universidad Católica de Valparaíso, 2340000 Valparaíso, Chile; byron.morales@pucv.cl (B.M.-L.); paulina.schmitt@pucv.cl (P.S.); 2 Instituto de Biología Molecular y Celular (IBMC) and Instituto de Investigación, Desarrollo e Innovación en Biotecnología Sanitaria de Elche (IDiBE), Universidad Miguel Hernández (UMH), 03202 Elche, Spain; ivan.nombela@kuleuven.be (I.N.); mortega-villaizan@umh.es (M.D.M.O.-V.); 3 Laboratory for Molecular Virology and Gene Therapy, Department of Pharmaceutical and Pharmacological Sciences, KU Leuven, Leuven, 3000 Flanders, Belgium; 4 Centro de Biotecnología Acuícola, Departamento de Biología, Universidad de Santiago de Chile, Estación Central, 9160000 Santiago, Chile; monica.imarai@usach.cl

**Keywords:** *Oncorhynchus mykiss*, mononuclear cells, interferon-gamma, transcriptional factors, *Piscirickettsia salmonis*

## Abstract

**Simple Summary:**

In aquatic biological models, the communication between cells from the immune system remains poorly characterized. In this work, to determine the gene expression of master transcriptional factors that coordinate the polarization of T cells, co-cultures of rainbow trout splenocytes are analyzed after stimulation with Interferon-gamma and/or *Piscirickettsia salmonis*. The results showed an upregulation of *foxp3* compared to the other transcriptional factors, suggesting a potential communication between cells in the spleen, which may induce a Treg phenotype.

**Abstract:**

In fish, the spleen is one of the major immune organs in the animal, and the splenocytes could play a key role in the activation and modulation of the immune response, both innate and adaptive. However, the crosstalk between different types of immune cells in the spleen has been poorly understood. In this work, an in vitro strategy is carried out to obtain and characterize mononuclear splenocytes from rainbow trout, using biomarkers associated with lymphocytes (CD4 and IgM) and antigen-presenting cells (CD83 and MHC II). Using these splenocytes, co-cultures of 24 and 48 h are used to determine the gene expression of master transcriptional factors that coordinate the polarization of T cells (*t-bet*, *gata3*, and *foxp3*). The results show a proportional upregulation of *foxp3* (compared to *t-bet* and *gata3*) in co-cultures (at 24 h) of IFNγ-induced splenocytes with and without stimulation of *Piscirickettsia salmonis* proteins. In addition, *foxp3* upregulation was established in co-cultures with IFNγ-induced cells and in cells only stimulated previously with *P. salmonis* proteins at 48 h of co-culture. These results show a potential communication between antigen-presenting-like cells and lymphocyte in the spleen, which could be induced towards a Treg phenotype.

## 1. Introduction

Rainbow trout (*Oncorhynchus mykiss*) is one of the most important salmonids species with economic interest for the Aquaculture [1]. However, the maintenance of high production densities, may trigger an increase in the risk of infections and diseases in salmonids fish farms [2]. That is why the characterization of the immune system becomes important for the production process, because the main function of the immunity is to maintain the homeostasis in the animal when it is invaded by foreign compounds or organisms [3].

In teleost, the spleen has been considered a central component of the immune system, due to this organ plays an important role in responses against pathogen invasion [4]. The spleen is made up of a system of splenic ellipsoids, melanomacrophage centers, and lymphoid tissue. Mature T cells are distributed throughout this organ, and cells along the walls are actively involved in the antigens capture [5,6].

T cells can act in the orchestration of immune responses and consist of several cell subtypes with distinct functions [7]. In higher vertebrates, naïve T cells, in response to stimulation by Antigen Presenting Cells (APCs) and cytokines, can differentiate into different subpopulations, including Th1 (related to cell-mediated immunity), Th2 (mediator of antibody production), Th17 (associated with pro-inflammatory responses), or Treg (associated with immunosuppression) [8]. The differentiation and function of each T cell subset are controlled by specific master transcription factors [9], such as: T-box transcription factor (T-bet), which controls the progression to the Th1 phenotype, Trans-acting T cell-specific transcription factor GATA-3 (Gata3), which is essential for the differentiation of the Th2 subtype, RAR-related orphan receptor gamma (RORγt) that regulates the activation of subtypes Th17 and Forkhead box P3 (Foxp3) which is crucial for the differentiation to regulatory lymphocytes (Treg) [8]. In fish, lymphocytes are also key cells for adaptive immunity [10], and T cell master transcriptional factors (T-bet, Gata3, and Foxp3) have already been described with functions similar to those of higher vertebrates [9].

In higher vertebrates, interferon gamma (IFNγ) is a molecule that modulates the antigen presentation process and the polarization of T cells [11,12]. In mammalian Dendritic cells (DC), IFNγ can induce overexpression of surface cell markers (CD40, CD80, CD86, MHC-I, and MHC-II) and cytokines, such as IL-12, which coordinate the signaling pathways necessary for an efficient antigen presentation, which finally activates CD4^+^ and CD8^+^ T cells [13,14].

In trout, it has been reported that IFNγ induces a higher level of APC-related markers (MHC-I and MHC-II), as well as down-regulates the expression of ZBTB46 [15], a transcriptional factor and repressor of the maturity of Antigen Presenting Cells (APCs) in mammals [16]. However, there is no evidence about the antigen-presenting cells inducing the Th cells polarization in salmonids and its modulation by IFNγ.

Based on this background, we have used splenocytes from *O. mykiss*, stimulated with IFNγ and an extract of proteins from the pathogen *Piscirickettsia salmonis* to achieve the modulation on gene expression of T cell master transcriptional factors. The mononuclear splenocytes, including MHCII^+^CD83^+^ cells, were co-cultured with sorted CD4^+^IgM^−^ cells from the spleen of rainbow trout to determine the gene expression of *t-bet*, *gata3*, and *foxp3*.

## 2. Materials and Methods

### 2.1. Fish

Thirty rainbow trout individuals of approximately 30 g. were obtained from a commercial fish farm (Piszolla S.L., Cimballa Fish Farm, Zaragoza, Spain). Fish were maintained in facilities with recirculating water systems belonging to the University Miguel Hernandez (UMH). Animals were fed daily with a commercial diet (Skretting, Burgos, Spain), and the water temperature was maintained at 14 °C. Fish were acclimatized to laboratory conditions for two weeks before experimentation. Experimental protocols and methods were approved by the Animal Welfare Body and the Research Ethics Committee at the UMH (2014.205.E.OEP and 2016.221.E.OEP) and by the Regional Ministry of Presidency and Agriculture, Fisheries, Food and Water supply (2014/VSC/PEA/00205). All methods were carried out in accordance with the Spanish Royal Decree RD 53/2013 and EU Directive 2010/63/EU for the protection of animals used for research experimentation.

### 2.2. Splenocytes

From each fish, the spleen was removed under aseptic conditions and broken on a nylon mesh (100 μm^2^) with an L15 culture medium (Gibco). Thereafter, 1 mL of each solution was added in a 2 mL ficoll gradient. All gradients were centrifuged at 180× *g* for 30 min at 14 °C. The mononuclear fraction was recovered and washed in a 15 mL tube with L15 medium. Then, the tubes were centrifuged at 180× *g* for 7 min at 14 °C, and the supernatant was removed. Cells were resuspended in 1 mL of L15 medium and viability and cell count and were performed by the Trypan blue method. All samples displayed cell viability higher than 95%. Finally, the cells were resuspended in a complete L15 medium (10% FBS and 1% penicillin-streptomycin). From each fish, 5 × 10^5^ cells were seeded per well (in triplicated) in 24-well plate. Cells were stabilized for 2 h, after that, non-adherent cells were removed. Non-adherent cells were cultivated for 15 days with a complete L15 medium. While, on the other hand, a complete L15 medium was added in adherent cells for overnight incubation. The next day, adherent cells were divided into four experimental conditions: (1) Control without induction (with fifty µL of sterile PBS 1x). (2) Splenocytes induced with IFNγ for 15 days (100 ng mL^−1^ of rIFNγ [17] suspended in sterile PBS 1x). (3) Splenocytes induced with 100 ng mL^−1^ of total protein extract of *Piscirickettsia salmonis* EM90 in PBS 1x [18] for 2 days (starting on day 13 of cell culture) as an antigen. (4) Splenocytes induced with IFNγ and *P. salmonis* (same concentration and times described above).

### 2.3. Immunofluorescence and Flow Cytometry

Immunofluorescence and flow cytometry analyses were carried out in splenocytes, cultured for 15 days from six fish (adherent cells with and without rIFNγ and non-adherent cells, separately).

For immunofluorescence, cells were washed with PBS 1x and seeded with PBS 1x + 0.1% FBS in 24 wells plate pre-treated with Poly-Lysine. Blocking was performed with 5% BSA for 30 min. Then, primary antibodies (Table 1) diluted in BSA 1% were incubated for 60 min at room temperature. After three washes with PBS 1x, cells were incubated with a commercial secondary antibody (Thermo Fischer Scientific Inc. Carlsbad, CA, USA) diluted 1:200 in BSA 1% (anti-mouse IgG Alexa 488 and anti-rabbit IgG Alexa 647) for 60 min at room temperature. Finally, the cells were washed with PBS 1x and fixed with 4% paraformaldehyde (in PBS 1x) for 10 min. For image capture, cell plates were placed in an IN Cell Analyzer 2200 (GE Healthcare, Little Chalfont, UK).

For flow cytometry, cells were recovered in IF medium (2% FBS in PBS) and centrifuged at 180× *g* for 7 min at 18 °C. Then, cells were resuspended in 1 mL of IF medium and incubated with the primary antibody (CD4-1 or CD83, Table 1) for 1 h at room temperature. Later, cells were washed, centrifuged, and incubated with the secondary antibody (anti-rabbit IgG-Alexa 488) for 1 h at room temperature. Finally, cells were fixed with 4% paraformaldehyde and resuspended in IF at room temperature in the dark. Flow cytometry was performed in a FACSJazz (BD Biosciences, Madrid, Spain).

### 2.4. Co-Culture of Splenocytes from Rainbow Trout

For co-culture assays, 15-day splenocytes from twelve fish per co-culture time were used (three fish per experimental condition). Briefly, cell count and viability assay (by Trypan blue) was performed both in adherent splenocytes, cultured for 15 days, as well as in non-adherent cells after being sorted by FACS-cell-sorting, applying the criteria of size, cellular complexity (associated lymphocytes) and negativity to the IgM marker (Table 1). All samples displayed cell viability between 88% to 94%. Then, 1 × 10^5^ adherent splenocytes the different experimental conditions were co-cultured in 24-well plates (in triplicated) for 24 and 48 h in ratio 1:1 with 1 × 10^5^ sorted non-adherent mononuclear splenocytes (CD4^+^ > 98% by immunofluorescence) from the same fish.

### 2.5. Gene Expression

Using the co-cultured splenocytes samples, E.Z.N.A. Total RNA Kit (Omega Bio-Tek Inc., Norcross, GA, USA) was used for total RNA extraction following the supplier’s instructions. To eliminate possible residual genomic DNA, all samples were treated using TURBO™ DNase (Ambion, Thermo Fischer Scientific Inc., Carlsbad, CA, USA) [20]. Total RNA was quantified using a NanoDrop Spectrophotometer (Nanodrop Technologies, Wilmington, DE, USA). cDNA was synthesized from total RNA using M-MLV reverse transcriptase (Invitrogen, Thermo Fischer) [21]. cDNA was stored at −80 °C until use.

For quantitative reverse transcription PCR (RT-qPCR), specific primers were designed to amplify *t-bet*, *gata3*, *foxp3* (Table 2). As a housekeeping gene, only e*f1α* was used (Table 2). RT-qPCR was carried out using 15 µL reaction mixtures containing Brilliant II SYBR^®^ Green QPCR MM (Agilent Technologies, Santa Clara, CA, USA), 0.2 µM of each primer, and 12 ng of cDNA diluted in sterile ultra-pure water. RT-qPCR assays were performed in triplicate for each gene per sample in an ABI PRISM 7300 System (Thermo Fischer Scientific). Cycling conditions were 50 °C for 2 min; 95 °C for 10 min; and 40 cycles of 95 °C for 15 s and 60 °C for 1 min. Primer pair efficiencies were calculated from seven serial dilutions of pooled cDNA for each primer pair according to the equation: E = 10[−1/slope] (Supplementary Figure S1). 

Relative expression was calculated using the 2^−ΔΔCt^ method [22], using the measured quantification cycle (Cq) values of ef1α housekeeping gene to normalize the measured Cq values of target genes.

### 2.6. Statistical Analysis

GraphPad v7.03 software was used to calculate estimated means, standard deviation, One-way ANOVA, and Tukey test for multiple comparisons. For correlation coefficients, corrplot package in R (available on CRAN: http://cran.r-project.org/package=corrplot (accessed on 2 March 2021) was used. Differences were considered significant when the *p*-value was < 0.05. Flow cytometry results were plotted using FlowJo v10.

## 3. Results and Discussion

### 3.1. Rainbow Trout Splenocytes

**Figure 1:**
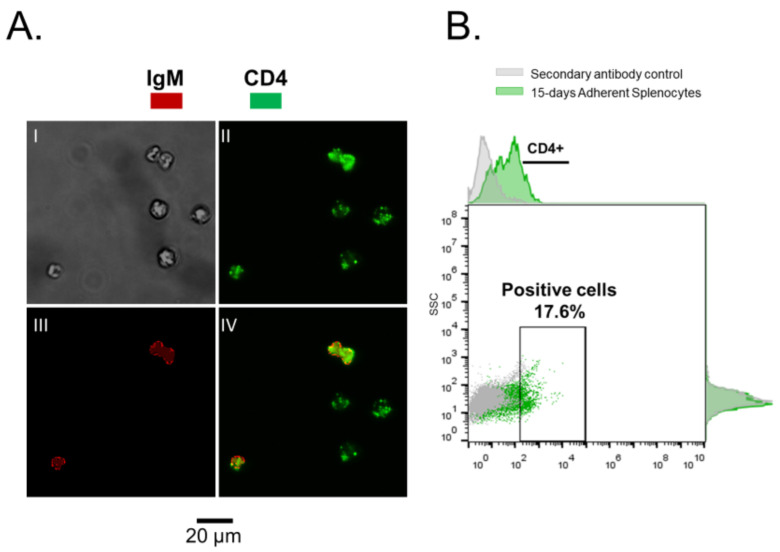
Non-adherent splenocytes, cultured for 15 days. (**A**). Immunofluorescence (IFAT) on mononuclear splenocytes, I—phase contrast, II—in green CD4, III—in red IgM, IV—Merged. (**B**). CD4^+^ cells. In gray—secondary antibody control. In green—non-Adherent Splenocytes.

In teleost fish, the spleen is one of the most important immune organ [4], and it is composed for a system of splenic ellipsoids, melanomacrophage centers, and lymphoid tissue with T cells (which are distributed throughout the organ) and other immune cells (along the walls) that can be actively involved in the antigens capture [5,6]. In this work, using rainbow trout as a biological model, 17.6% of total non-adherent splenocytes were detected as CD4^+^ after 15 days of culture (Figure 1). However, although these cells showed a positive label for CD4 alone, some cells were also double positive for CD4 and IgM (co-localization) (Figure 1). These results agree with those proposed by Maisey et al. [19]. In that study, authors have determined a subpopulation of CD4^+^ cells in the spleen of rainbow trout at the phenotypic level (CD4^+^ cells: 18.5%), where some of which can overexpress IgM at the same time.

In higher vertebrates, CD4^+^ T cells play a central role in immune protection, being that CD4^+^ T cells can orchestrate the full panoply of immune responses, because they have the capacity to induce B cells to make antibodies, and they also can stimulate macrophages to develop enhanced microbicidal activity, through their production of cytokines and chemokines [23]. In fish, CD4^+^ T cells are vital players in the immune response through different effector subtypes, such as Th1, Th2, or Treg. These T cell subtypes participate in stimulating macrophages (improving cell-mediated immunity), increasing the activity of B cells to produce antibodies, and regulating the immune response by immunosuppression [23,24].

**Figure 2:**
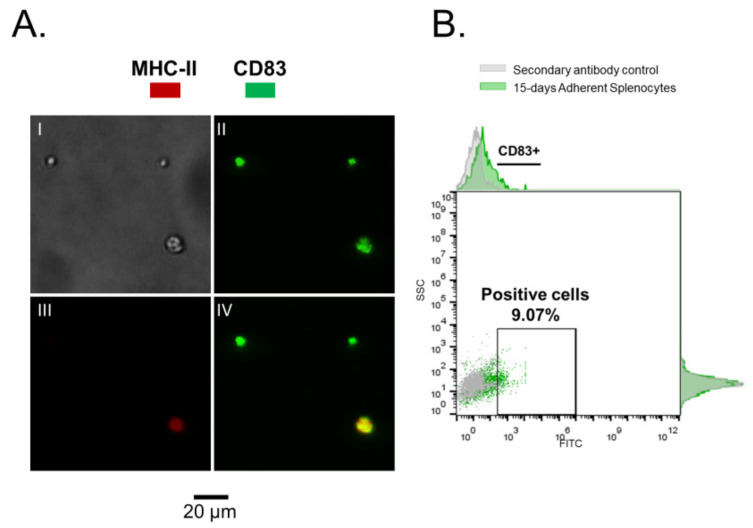
Adherent splenocytes, cultured for 15 days without rIFNγ. (**A**). Immunofluorescence (IFAT) on mononuclear splenocytes, I—phase contrast, II—in green CD83, III—in red MHC II, IV—Merged. (**B**). CD83^+^ cells. In gray—secondary antibody control. In green—adherent Splenocytes w/o rIFNγ.

**Figure 3:**
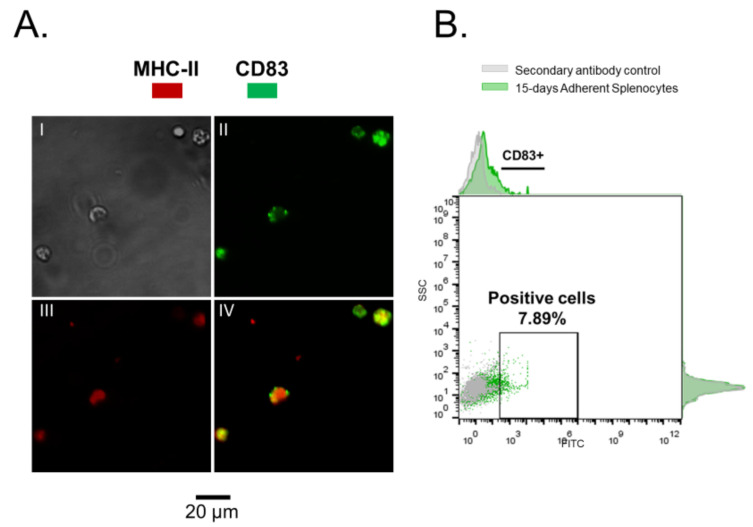
Adherent splenocytes, cultured for 15 days with rIFNγ as inducer. (**A**). Immunofluorescence (IFAT) on mononuclear splenocytes, I—phase contrast, II—in green CD83, III—in red MHC II, IV—Merged. (**B**). CD83^+^ cells. In gray—secondary antibody control. In green—adherent Splenocytes with rIFNγ.

In addition to CD4^+^ T cells, adherent splenocytes were recovered from primary cultures after 15 days with and without IFNγ induction, showing a positive detection for CD83 (Figure 2 and Figure 3) for a small cell-subpopulation (w/rIFNγ: 7.89% and w/o rIFNγ: 9.07%, respectively). In mammals, CD83 is a marker of mature dendritic cells and is related to the processes of antigen presentation. CD83 functions as an immuno-regulator protein, inducing immune responses mediated by T cells, delivering co-stimulatory signals that activate T lymphocytes, and stimulating their proliferation [25,26].

In salmonids, CD83 has already been described in cells with antigen presentation function during infectious pathogens [20,27]. Likewise, adherent splenocytes obtained in the present study also expressed MHC-II (Figure 1B). MHC-II is a cell-surface marker, and it primarily resides on the surface of professional antigen-presenting cells and can act by binding and presenting peptides from exogenously derived proteins to CD4^+^ T cells [28]. In rainbow trout, Nombela et al. [20] has already reported cells that overexpress MHC-II during the antigen presentation process. Similarly, Wang et al. [15] described that the induction with IFNγ induced a higher level of MHC-I and MHC-II in macrophages from rainbow trout.

### 3.2. Gene Expression of Transcriptional Factors Related to T Cell Polarization

**Figure 4:**
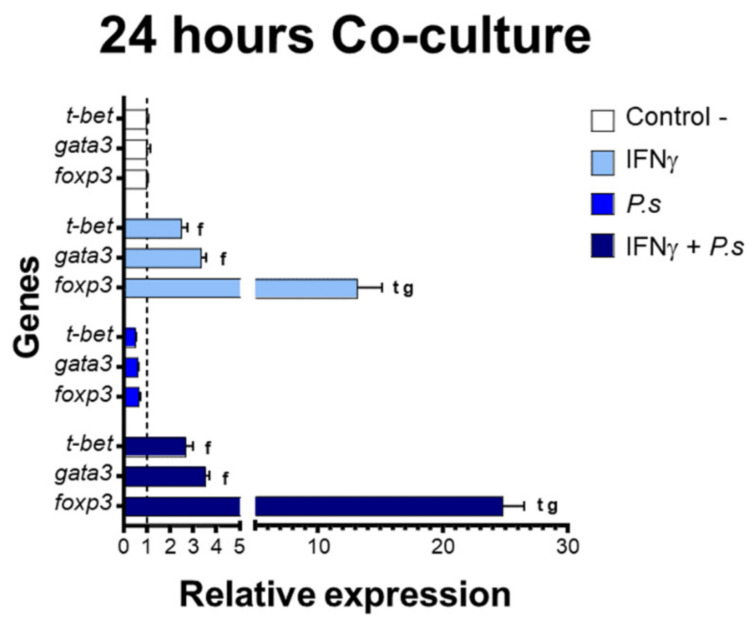
Gene expression presented as fold change relative to controlling T cell transcriptional factors in co-cultures of CD4^+^ IgM^−^ cells and mononuclear splenocytes MHC^+^ CD83^+^, that include MHC^−^ CD83^−^ cells at 24 h. In white—control without induction (C-). In light blue—co-culture of splenocytes with prior induction with IFNγ. In blue—co-culture of splenocytes with prior induction of *P. salmonis* (*P.s*). In dark blue—co-culture of splenocytes induced with IFNγ + *P. salmonis* (IFNγ+*P.s*). Each bar: *n* = 3 fish. Lowercase letters (t, g, and f)—significant differences (*p* < 0.05) compared with *t-bet*, *gata3*, and *foxp3*, respectively.

**Figure 5:**
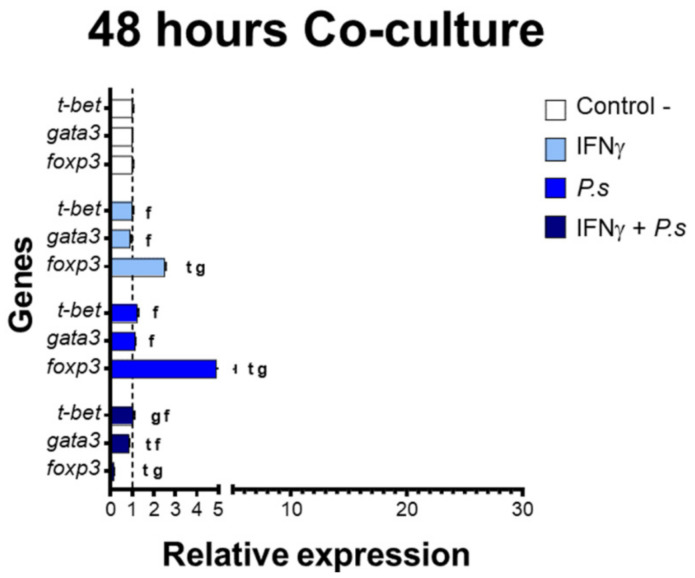
Gene expression presented as fold change relative to controlling T cell transcriptional factors in co-cultures of CD4^+^ IgM^−^ cells and mononuclear splenocytes MHC^+^ CD83^+^, that include MHC^−^ CD83^−^ cells at 48 h. In white—control without induction (C-). In light blue—co-culture of splenocytes with prior induction with IFNγ. In blue—co-culture of splenocytes with prior induction of *P. salmonis* (*P.s*). In dark blue—co-culture of splenocytes induced with IFNγ + *P. salmonis* (IFNγ+*P.s*). Each bar: *n* = 3 fish. Lowercase letters (t, g, and f)—significant differences (*p* < 0.05) compared with *t-bet*, *gata3*, and *foxp3*, respectively.

**Figure 6:**
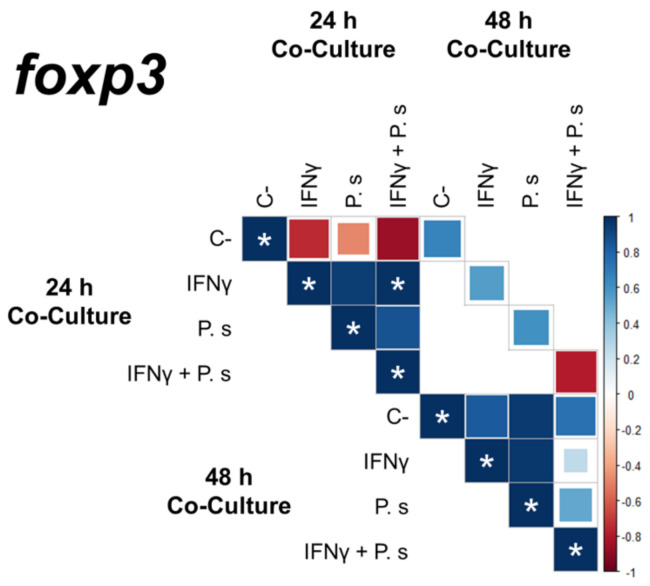
Correlation coefficient of *foxp3* gene expression in co-cultures of CD4^+^ IgM^−^ cells and adherent splenocytes (MHCII^+^ CD83^+^/MHCII^−^ CD83^−^) cells at 24 and 48 h. C-—control without induction. IFNγ—splenocytes cells induced with IFNγ. P.s—splenocytes induced with *P. salmonis*. IFNγ + P.s—splenocytes induced with IFNγ and *P. salmonis*. * significant value (*p* < 0.05).

The gene expression of transcriptional factors of T cell polarization was assessed from co-cultures of sorted CD4^+^ IgM^-^ cells and IFNγ-induced mononuclear splenocytes, including MHCII^+^ CD83^+^ cells, with and without stimulation of *P. salmonis* proteins (Figure 4 and Figure 5). Expression results from 24 h co-cultures (Figure 4) showed a significant up-regulation of *fopx3* (IFNγ: 13.18-fold, IFNγ + *P. salmonis*: 24.82-fold) compared to the expression of *t-bet* (IFNγ: 2.48-fold, IFNγ + *P. salmonis*: 2.68-fold) and *gata3* (IFNγ: 3.33-fold, IFNγ + *P. salmonis*: 3.52-fold). Additionally, the increase in *foxp3* expression showed a positive correlation between the co-cultures that had IFNγ-induced splenocytes with and without stimulation of *P. salmonis* proteins (Figure 6). These data are based on the notion that the modulation of transcription factors related to T cell polarization occurs in CD4^+^ cells. In higher vertebrates, T cells must crosstalk with APCs to induce the expression of transcription factors to differentiate into effector subtypes [29]. In here, both MHC^+/−^ and CD83^+/−^ splenocytes are present at the co-culture assay; however only MHCII^+^ CD83^+^ splenocytes could interact with CD4^+^ cells.

In parallel, a lower expression of transcriptional factors of T cell polarization was detected at 48 h of co-culture (Figure 5). Despite this, a significant upregulation of *foxp3* was determined compared to *t*-*bet* and *gata3* expression in co-cultures with IFNγ-induced splenocytes (*foxp3*: 2.47-fold, *t-bet*: 0.98-fold and *gata3*: 0.92-fold) and in those that only had previous stimulation with *P. salmonis* proteins (*foxp3*: 4.90-fold, *t-bet*: 1.24-fold and *gata3*: 1.15-fold). On the other hand, in co-cultures with IFNγ-induced splenocytes and *P. salmonis* proteins, a downregulation of *fopx3* (0.15-fold) was established when compared to *t-bet* (1.04-fold) and *gata3* (0.83-fold).

In fish, the transcriptional factors t-bet, gata3, and fopx3 have already been reported as those capable of governing and modulating the activation and polarization of T cells to effector subtypes [9]. T-bet is a master transcriptional factor associated with the Th1 profile capable of coordinating the immune response against intracellular bacterial infections [8], which has been described in rainbow trout during bacterial and parasitic infections [30]. Regarding gata3, this molecule is also a master transcriptional factor, but it is associated with Th2 phenotype. Th2 cells are involved in the modulation of B cells (by cytokines, such as IL-4) to produce immunoglobulins [9]. Finally, foxp3 is a transcriptional factor related to regulatory profiles (Treg), modulating different processes, such as immunosuppression and homeostasis. [8,31].

It should be noted that the overexpression of foxp3 detected in the present study occurred in co-cultures between CD4^+^ IgM^-^ cells and IFNγ-induced mononuclear splenocytes, including MHCII^+^ CD83^+^ cells, with or without *P. salmonis* proteins. The nature of these stimulatory molecules projected a more marked induction of t-bet and/or gata3, which are related to the activation of cell-mediated immunity and with the production of antibodies, respectively [9,30], and not an increase of fopx3, which is related to immunosuppression profiles [31]. In mammals, IFNγ can act by modulating both the innate and adaptive immune responses, regulating the maturation processes of antigen-presenting cells, and activating CD4^+^ T cells to Th1 subtype [13,14,32]. In fish, it has also been proposed that IFNγ would induce responses associated with Th1 profiles crucial for coordinating the host response to intracellular pathogens [33,34], such as *P. salmonis*. Therefore, we hypothesized that the upregulation of foxp3 after stimulation with IFNγ and *P. salmonis* proteins could be associated with the fact that splenocytes modulate aspects related to their homeostasis. Reyes-López et al. [35] described in fish that the immune response must be implemented through integrated and regulated pathways, since abrupt and exacerbated responses to a pathogen can promote the death of the host that fights infection. In addition, in higher vertebrates, the immunosuppressive function of fopx3 is both related to the maintenance of homeostasis, as well as to the survival of memory T cells [28]. Memory cells are crucial for a secondary activation of the immune response. Also, they are the final target for the success of vaccination strategies, which are widely used in aquaculture. Nevertheless, these strategies have not yet been able to generate an improved response, over time, against pathogens, such as *P. salmonis* [36]. Using a cyprinid study model, Piazzon et al. [37] have proposed that phenotypic environments associated with regulatory lymphocytes not only generate the suppression of immunity, but could also be related to the maintenance of memory cells, over time. Overall, further studies on memory T cells in salmonids must be performed as they remain poorly characterized.

In non-mammalian vertebrates, the studies that characterize the functions of Treg and foxp3 are few. However, this molecule may have an evolutionally conserved role in controlling immune tolerance in fish [38]. Interestingly, in recent years it has been proposed that other immune cells, such as macrophages and B cells (from humans and mice), could express foxp3; nevertheless, this proposal is still a matter of controversy [39,40]. Regarding fish, in Nile tilapia (*Oreochromis niloticus*), Jia et al. [41] reported that although foxp3 is mainly expressed in lymphatic tissues, it could also be detected in epithelial cells of organs not commonly associated with the immune response (stomach). In salmonids, future studies should address whether foxp3 can be expressed in different cell types in organs related or not to the immune system, this will allow a better understanding of how the responses associated with immunosuppression and fish homeostasis are coordinated, which could be crucial for maintaining high production densities of economically important fish.

## 4. Conclusions

Co-cultures from rainbow trout splenocytes (CD4^+^IgM^−^ cells and IFNγ-induced cells, including MHC^+^CD83^+^) with or without *P. salmonis* proteins, can trigger an increase in the expression of *foxp3*, associated with the polarization of T cells to Treg phenotype.

## Figures and Tables

**Table 1:** Primary antibodies used in IFAT and FACS.

Molecule	Type	Source	Dilution	Reference
CD4-1	IgG Polyclonal	Rabbit	1:200	[19]
CD83	IgG Polyclonal	Rabbit	1:100	[20]
IgM	IgG Monoclonal	Mouse	1:100	[19]
MHC-II	IgG Polyclonal	Mouse	1:200	[20]

**Table 2:** List of primers used for gene amplification by RT-qPCR.

Gene	Primer	Sequence	Tm	Reference (GenBank)
t-bet	Forward	GAAGTGAAGGAGGATGGTTCTG	55 °C	GU979861.1
	Reverse	GTGATGTCTGCGTTCTGATAGG	55 °C	
gata3	Forward	CGACGATGTGGATGTACTGTTTA	55 °C	EU418015.1
	Reverse	TACTATGTGGAGGAGGTGGATAC	55 °C	
foxp3	Forward	AGACTGAGAGTGAGAGGTTGA	55 °C	HQ270469.1
	Reverse	TAGAGCCGAGGTGAGAAAGA	55 °C	
ef1α	Forward	TGGAGACTGGCACCCTGAAG	55 °C	AF498320.1
	Reverse	CCAACATTGTCACCAGGCATGG	55 °C	

## Data Availability

The data presented in this study are available on request from the corresponding author.

## Supplementary Materials

The following are available online at https://www.mdpi.com/article/10.3390/biology10040324/s1, Figure S1: Primer efficiency (%) per gene.
