# Supplementary figures and images for: Induction of foxp3 during the Crosstalk between Antigen Presenting Like-Cells MHCII+CD83+ and Splenocytes CD4+IgM− in Rainbow Trout
Source: Biology (Basel). 2021 Apr 13;10(4):324. doi: 10.3390/biology10040324 (PMC8069158; doi:10.3390/biology10040324)

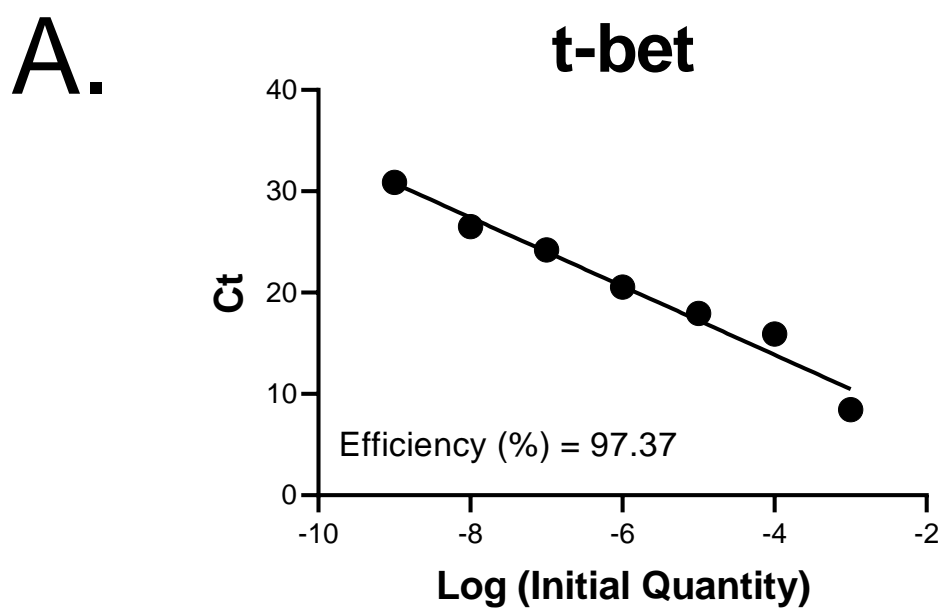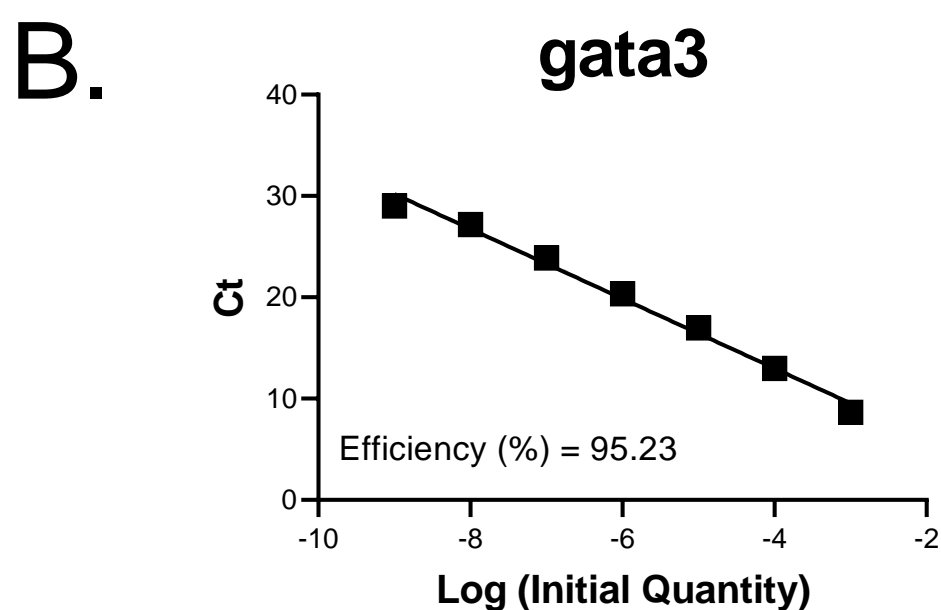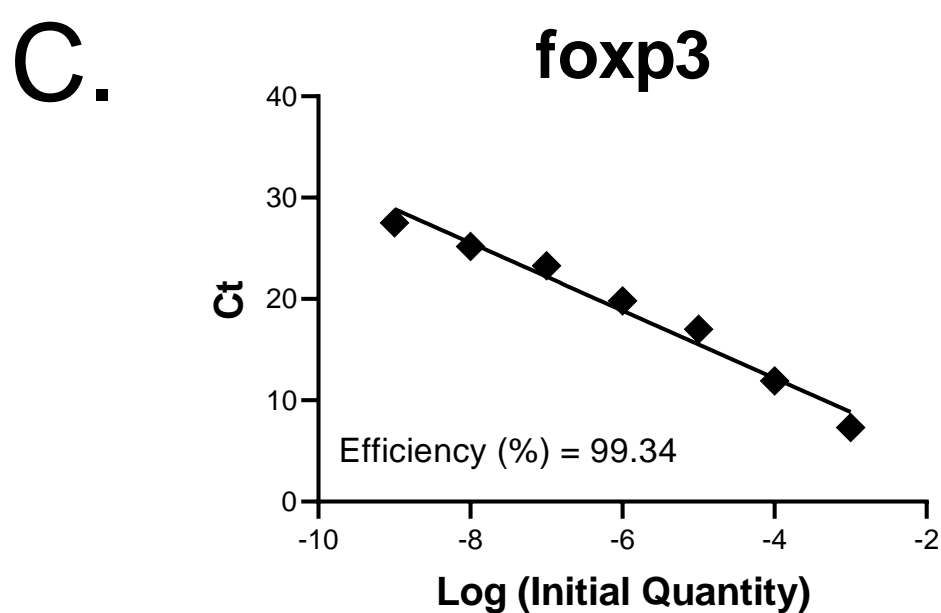

**Supplementary figure 1.** Primer efficiency (%) per gene. A: t-bet. B: gata3. C: foxp3.

Supplement: Supplementary file 1 [file biology-10-00324-s001.pdf]
